# HIV Stigma and Health Care Discrimination Experienced by Hispanic or Latino Persons with HIV — United States, 2018–2020

**DOI:** 10.15585/mmwr.mm7141a1

**Published:** 2022-10-14

**Authors:** Mabel Padilla, Deesha Patel, Linda Beer, Yunfeng Tie, Priya Nair, Yamir Salabarría-Peña, Kirk D. Henny, Dominique Thomas, Sharoda Dasgupta

**Affiliations:** ^1^Division of HIV Prevention, National Center for HIV, Viral Hepatitis, STD, and TB Prevention, CDC; ^2^DLH Corporation, Atlanta, Georgia.

Hispanic or Latino (Hispanic) persons with HIV experience disparities in HIV health outcomes compared with some other racial and ethnic groups. A previous report found that the percentages of Hispanic persons who received HIV care, were retained in care, and were virally suppressed were lower than those among non-Hispanic White persons with HIV (*1*). HIV stigma and discrimination are human rights issues associated with adverse HIV outcomes; eliminating stigma and discrimination among persons with HIV is a national priority*^,^^†^^,^^§^ (*2*,*3*). CDC analyzed data from the Medical Monitoring Project (MMP), an annual, cross-sectional study designed to report nationally representative estimates of experiences and outcomes among adults with diagnosed HIV. Data from the 2018–2020 cycles were analyzed to assess self-reported stigma and health care discrimination using adapted versions of validated multi-component scales among 2,690 adult Hispanic persons with HIV in the United States overall and by six characteristics.^¶^ The median HIV stigma score on a scale of 0–100 was 31.7, with women (35.6) and American Indian or Alaska Native (AI/AN) persons (38.9) reporting the highest scores among Hispanic persons with HIV. HIV stigma was primarily attributed to disclosure concerns (e.g., fearing others will disclose one’s HIV status and being careful about who one tells about one’s HIV status). Nearly one in four (23%) Hispanic persons with HIV experienced health care discrimination. Health care discrimination was experienced more frequently by Hispanic men (23%) than by Hispanic women (18%) and by Black or African American (Black) Hispanic persons (28%) than by White Hispanic persons (21%). Understanding disparities in experiences of stigma and discrimination is important when designing culturally appropriate interventions to reduce stigma and discrimination.

MMP uses a two-stage sampling method. First, in 2004, out of all U.S. states, the District of Columbia, and Puerto Rico, 16 states and Puerto Rico were sampled**^,^^††^ (*4*). Second, a simple random sample of adults with diagnosed HIV is selected annually from each participating jurisdiction in the National HIV Surveillance System (NHSS), a census of persons with diagnosed HIV in the United States. During the 2018–2020 data cycles, data were collected through telephone or in-person interviews. Response rates for the two data cycles were 100% (jurisdictions) and 40%–45% (individual respondents). HIV stigma was measured using an adapted version of a validated 10-item scale that measures four dimensions of HIV stigma: 1) personalized stigma (consequences of other people knowing their status), 2) disclosure concerns, 3) negative self-image (not feeling as good as others and experiencing shame or guilt), and 4) public attitudes (what people think about HIV)^§§^ (*5*). Responses (strongly disagree, somewhat disagree, neutral, somewhat agree, and strongly agree) for each item were given scores of 0, 2.5, 5, 7.5, and 10, respectively, and summed to a score ranging from zero (no stigma) to 100 (high stigma). HIV health care discrimination during the previous 12 months was assessed based on seven forms of discrimination, using an adapted version of a validated Likert scale^¶¶^ (*6*). Participants who reported experiencing at least one form of health care discrimination were considered to have experienced discrimination in an HIV health care setting; those who experienced any discrimination were asked whether they attributed discrimination to any of six characteristics.***

HIV stigma and health care discrimination were assessed overall and by the following demographic characteristics: gender, race,^†††^ Hispanic origin,^§§§^ country or region of birth,^¶¶¶^ and English proficiency.**** Analyses were weighted to adjust for individual nonresponse and poststratified to match the actual number of persons with diagnosed HIV in NHSS by age, race and ethnicity, and gender. Median scores and 95% CIs (using t distribution) were calculated to assess HIV stigma; nonoverlapping CIs determined meaningful differences among groups. Prevalence ratios (PRs) with predicted marginal means were used to quantify differences by characteristics; p<0.05 was considered statistically significant. All analyses were conducted using SAS (version 9.4; SAS Institute) and SAS-callable SUDAAN (version 11.0.1; RTI International). This activity was reviewed by CDC and was conducted consistent with applicable federal laws and CDC policy.^††††^

Among Hispanic persons with HIV (2,690), 81% were male, 66% identified as White, 13% identified as Black, and 4% identified as AI/AN (Table 1). Thirty-six percent identified Hispanic origin as Mexican, Mexican American, or Chicano; 34% identified Hispanic origin as Puerto Rican. Nearly two thirds (62%) were born outside the continental United States, 22% were born in Puerto Rico, and 19% in Mexico; 42% had limited English proficiency.

**TABLE 1:** Demographic characteristics of Hispanic or Latino adults with diagnosed HIV — Medical Monitoring Project, United States, 2018–2020

Characteristic*	No.^†^	Weighted % (95% CI)
**Overall**	**2,690**	—
**Gender** ^§^		
Male	2,043	80.8 (78.6–82.9)
Female	576	19.2 (17.1–21.4)
**Race^¶^**		
Asian	—**	—**
American Indian or Alaska Native	101	3.8 (2.8–4.9)
Black or African American	350	13.1 (10.1–16.1)
White	1,697	66.0 (61.9–70.1)
Multiple races	284	9.4 (7.7–11.2)
Race not selected	206	7.4 (5.9–8.9)
**Hispanic origin**		
Mexican, Mexican American, or Chicano	842	35.7 (28.7–42.6)
Puerto Rican	1,004	33.5 (22.7–44.3)
Cuban	79	3.2 (2.3–4.2)
Another Hispanic origin^††^	739	27.6 (23.4–31.7)
**Born outside the United States** ^§§^		
No	977	38.3 (31.9–44.6)
Yes	1,701	61.7 (55.4–68.1)
Puerto Rico	706	22.2 (9.3–35.1)
Mexico	467	19.0 (14.9–23.1)
Central America	186	7.5 (5.6–9.5)
South America	174	6.5 (4.9–8.1)
Caribbean (excludes Puerto Rico)	141	5.5 (4.5–6.6)
Another country or region	27	1.0 (0.5–1.4)
**Limited English proficiency** ^¶¶^		
Yes	885	41.7 (39.2–44.2)
No	1,257	58.3 (55.8–60.8)

* All variables measured by self-report.

^†^ Numbers might not add to total because of missing data.

^§^ Participants who identified as transgender were excluded from this analysis because of small sample sizes.

^¶^ Race and ethnicity were measured based on Office of Management and Budget Directive No.15. Participants were asked “Do you consider yourself to be of Hispanic, Latino/a, or Spanish origin?” and “Which racial group or groups do you consider yourself to be in? You may choose more than one option.” Hispanic or Latino (Hispanic) participants were categorized as White if they considered themselves to be White and said “no” to all other races. Asian, American Indian or Alaska Native, and Black or African American Hispanic persons were categorized similarly. Participants who answered “no” to all races, refused to identify with all of the races, or had some combination of these were classified as “race not selected.” Participants who selected more than one race were classified as “multiple races.”

** Data for Hispanic persons who identified as Asian are not included because of small sample sizes.

^††^ Participants who selected “another Hispanic, Latino/a, or Spanish origin” or multiple Hispanic or Spanish origins (e.g., Mexican, Puerto Rican, or Cuban) were categorized as “another Hispanic origin.”

^§§^ Persons born in Puerto Rico or another U.S. territory were categorized as being born outside the United States for the purpose of this analysis because of differences in cultural context.

^¶¶^ Participants who spoke English less than “very well” and spoke a language other than English at home were categorized as having limited English proficiency. Persons currently living in Puerto Rico were excluded from this variable because English is not the primary language spoken in Puerto Rico.

The overall median HIV stigma score among Hispanic persons with HIV was 31.7 (Table 2). HIV stigma was higher among Hispanic women (median = 35.6) than among Hispanic men (median = 30.3) and was also high among Hispanic persons with HIV who identified as AI/AN (median = 38.9) and those who were born in the Caribbean (median = 35.7) (Table 2). Disclosure concerns and perceived public attitudes about persons with HIV were the most reported HIV stigma domains. Forty-eight percent to 78% of persons with HIV strongly agreed with the two items about disclosure concerns, and 20%–28% strongly agreed with the two items about perceived public attitudes (Figure 1) (Supplementary Table 1, https://stacks.cdc.gov/view/cdc/121706).

**TABLE 2:** HIV stigma scores and prevalence of HIV health care discrimination experienced by Hispanic or Latino adults with diagnosed HIV, by selected characteristics — Medical Monitoring Project, United States, 2018–2020

Selected characteristic^§^	HIV stigma*		Experienced any health care discrimination^†^			
	No.^¶^	Median score (95% CI)**	No.^¶^	% (95% CI)**	Prevalence ratio (95% CI)	p-value
**Overall**	**2,535**	**31.7 (30.3–33.1)**	**574**	**22.6 (20.7–24.5)**	**NA**	**NA**
**Gender** ^††^						
Male	1,932	30.3 (28.7–31.8)	453	23.4 (21.2–25.6)	Ref	
Female	537	35.6 (33.5–37.7)	102	18.3 (14.7–21.8)	0.8 (0.6–1.0)	0.018
**Race** ^§§^						
Asian		—^¶¶^	—^¶¶^	—^¶¶^	—^¶¶^	—^¶¶^
American Indian or Alaska Native	97	38.9 (33.2–44.5)	26	24.7 (15.8–33.5)	1.2 (0.8–1.7)	0.389
Black or African American	336	32.7 (30.5–34.9)	85	27.7 (23.0–32.5)	1.3 (1.1–1.7)	0.010
White	1,604	30.4 (28.8–32.1)	343	20.8 (18.5–23.2)	Ref	
Multiple races	261	31.7 (28.6–34.8)	72	28.7 (22.3–35.0)	1.4 (1.1–1.7)	0.010
Race not selected	192	34.7 (30.0–39.5)	40	22.7 (15.6–29.7)	1.1 (0.8–1.5)	0.622
**Hispanic origin**						
Mexican, Mexican American, or Chicano	793	32.3 (30.1–34.4)	171	20.4 (17.2–23.6)	Ref	NA
Puerto Rican	956	33.0 (31.4–34.7)	215	23.7 (21.2–26.3)	1.2 (1.0–1.4)	0.118
Cuban	75	32.9 (28.0–37.7)	17	30.0 (16.5–43.5)	1.5 (0.9–2.4)	0.134
Another Hispanic origin***	697	29.2 (27.5–30.9)	167	23.2 (19.4–26.9)	1.1 (0.9–1.4)	0.258
**Born outside the United States** ^†††^						
Yes	1,608	31.0 (29.2–32.8)	330	20.2 (17.8–22.6)	0.8 (0.6–0.9)	0.002
No	926	32.9 (30.6–35.2)	243	26.5 (23.2–29.8)	Ref	NA
**Country or region of birth**						
United States	926	32.9 (30.6–35.2)	243	26.5 (23.2–29.8)	Ref	
Puerto Rico	673	32.7 (31.3–34.1)	154	23.9 (21.4–26.4)	0.9 (0.8–1.1)	0.196
Mexico	435	30.4 (27.7–33.0)	78	16.4 (12.3–20.5)	0.6 (0.5–0.8)	0.001
Central America	171	29.0 (26.8–31.2)	28	16.8 (10.6–23.0)	0.6 (0.4–0.9)	0.014
South America	170	26.7 (22.3–31.1)	33	16.7 (10.8–22.6)	0.6 (0.4–0.9)	0.012
Caribbean (excludes Puerto Rico)	132	35.7 (31.4–40.0)	26	22.0 (13.5–30.4)	0.8 (0.6–1.2)	0.352
Another country or region	27	27.3 (20.4–34.1)	11	—^§§§^	—^§§§^	—^§§§^
**Limited English proficiency** ^¶¶¶^						
Yes	830	32.4 (30.3–34.5)	141	16.6 (13.7–19.5)	0.6 (0.5–0.8)	<0.001
No	1,191	30.1 (28.2–31.9)	313	26.5 (23.4–29.5)	Ref	NA

**Abbreviations:** NA = not applicable; Ref = referent group.

* Range is from zero (no stigma) to 100 (high stigma).

^†^ During the previous 12 months.

^§^ All variables measured by self-report.

^¶^ Numbers are unweighted. Numbers might also not add to total because of missing data.

** Percentages are weighted row percentages, and CIs incorporate weighted percentages. All analyses were weighted to adjust for individual nonresponse and poststratified to match the actual number of persons with diagnosed HIV in National HIV Surveillance System (a census of persons with diagnosed HIV in the United States) by age, race and ethnicity, and sex.

^††^ Participants who identified as transgender were excluded because of small sample sizes.

^§§^ Race and ethnicity were measured based on Office of Management and Budget Directive No.15. Participants were asked “Do you consider yourself to be of Hispanic, Latino/a, or Spanish origin?” and “Which racial group or groups do you consider yourself to be in? You may choose more than one option.” Hispanic or Latino (Hispanic) participants were categorized as White if they considered themselves to be White and said “no” to all other races; Asian, American Indian or Alaska Native, and Black or African American Hispanic persons were categorized similarly. Participants who answered “no” to all races, refused to identify with all of the races, or had some combination of these were classified as “race not selected.” Participants who selected more than one race were classified as “multiple races.”

^¶¶^ Data not included because of small sample sizes.

*** Participants who selected “another Hispanic, Latino/a, or Spanish origin” or multiple Hispanic or Spanish origins (e.g., Mexican, Puerto Rican, or Cuban) were categorized as “another Hispanic origin.”

^†††^ Persons born in Puerto Rico or another U.S. territory were categorized as being born outside the United States for the purpose of this analysis because of differences in cultural context.

^§§§^ Estimates with a CI width ≥30 and those with an underlying denominator <30 were considered to be unstable and were therefore suppressed.

^¶¶¶^ Participants who spoke English less than “very well” and spoke a language other than English at home were categorized as having limited English proficiency. Persons currently living in Puerto Rico were excluded from this variable because English is not the primary language spoken in Puerto Rico.

**FIGURE 1:**
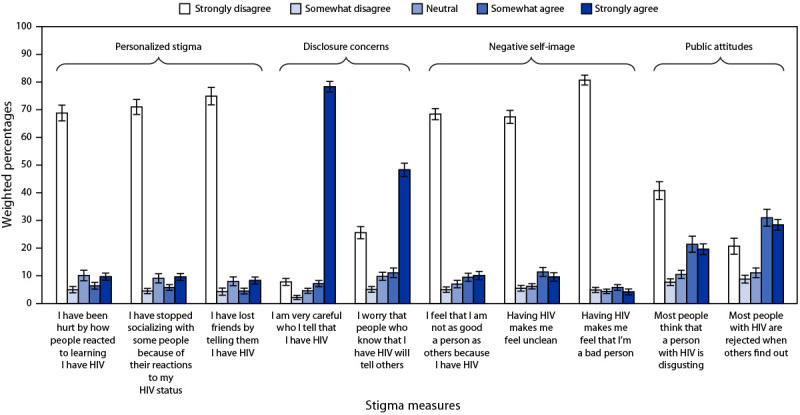
HIV stigma* reported by Hispanic or Latino adults with diagnosed HIV — Medical Monitoring Project, United States, 2018–2020 * Personalized stigma domain asked about the previous 12 months; other HIV stigma domains asked about current experiences of HIV stigma.

Overall, 22.6% of Hispanic persons with HIV reported experiencing any HIV health care discrimination during the previous 12 months (Table 2); 8% reported one, 4% reported two, and 11% reported three or more health care discrimination experiences (Supplementary Table 2, https://stacks.cdc.gov/view/cdc/121707). Among those who experienced health care discrimination, 62% felt that a doctor or nurse was not listening to what they were saying, 48% felt they were treated with less respect than others, and 48% perceived they were treated with less courtesy than others (Figure 2). Thirty percent attributed health care discrimination to their HIV infection, 23% to their sexual orientation or sexual practices, and 20% to their race or ethnicity (Figure 2).

**FIGURE 2:**
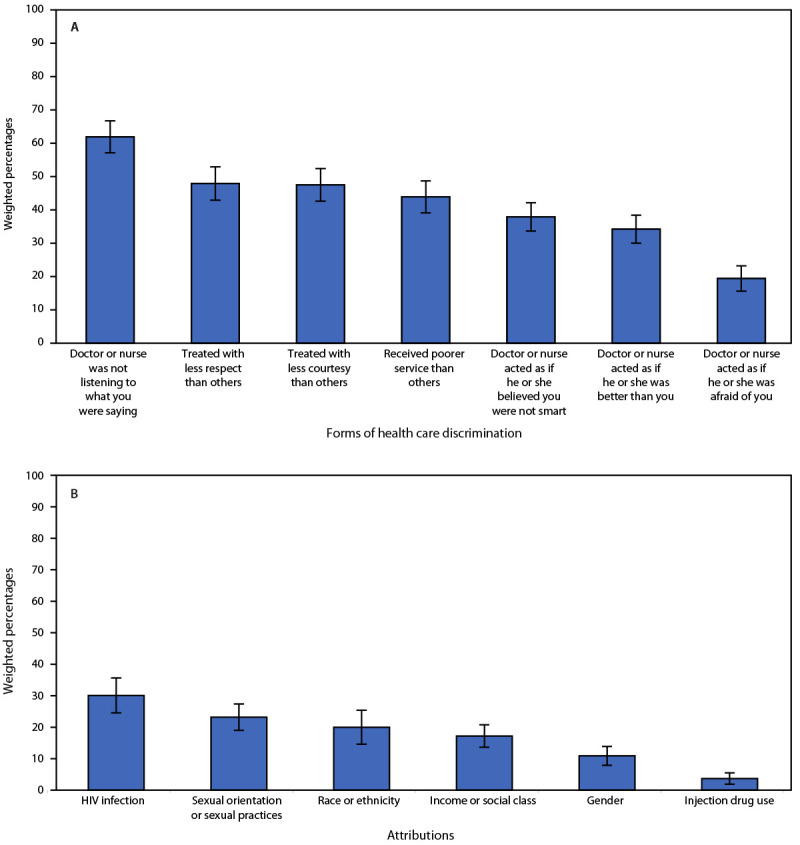
Forms of HIV health care discrimination*^,^^†^ (A) and attributions of HIV health care discrimination (B) reported by Hispanic or Latino adults with diagnosed HIV — Medical Monitoring Project, United States, 2018–2020 * 95% CIs indicated by error bars. ^†^ HIV health care discrimination experiences were measured during the previous 12 months.

Hispanic women were less likely to experience health care discrimination than were Hispanic men (PR = 0.8 Table 2). Black (PR = 1.3) and multiracial Hispanic persons were more likely than White Hispanic persons to experience health care discrimination (PR = 1.4). Non-U.S.–born persons (PR = 0.8) were less likely to experience health care discrimination than U.S.-born persons. Specifically, persons born in Mexico (PR = 0.6), Central America (PR = 0.6), and South America (PR = 0.6) were less likely to experience health care discrimination than U.S.-born persons. Persons with limited English proficiency (PR = 0.6) were less likely to experience health care discrimination than their counterparts.

## Discussion

HIV stigma and discrimination experiences in an HIV health care setting were commonly reported among Hispanic persons with HIV and varied by characteristics such as race, gender, and English proficiency. Hispanic persons with HIV are highly diverse. Efforts to reduce HIV stigma and discrimination should consider the varied and unique experiences of this population.

Similar to experiences reported by all U.S. persons with HIV, the most prevalent HIV stigma domain among Hispanic persons with HIV was concern about disclosure of HIV status (*2*), and the most reported form of health care discrimination was feeling that a clinician was not listening to them (*3*). This underscores the importance of addressing disclosure concerns when designing interventions to reduce HIV stigma. Training for providers should focus on actively listening to patient concerns, including stigma experiences, using culturally and linguistically appropriate methods.^§§§§^

Although HIV stigma was more commonly reported by Hispanic women than men, women experienced lower levels of health care discrimination. This contrasts with a study of Hispanic adults that found Latino men were less likely to report health care discrimination than women (*7*). The present study indicates that stigma and health care discrimination, although related, are distinct concepts experienced differently by Hispanic men and women. Given that more Hispanic men with HIV than women identified as gay or bisexual, these health care discrimination findings could also be based on sexual orientation.

Black Hispanic persons with HIV were more likely than White Hispanic persons with HIV to report health care discrimination. Though not equivalent to racial identity, some Hispanic persons use skin color to select a racial identity category. Health care discrimination experienced by Hispanic persons might differ based on skin color, with one study finding that Hispanic persons with darker skin experienced greater health care discrimination than those with lighter skin (*8*). Another study found that U.S.-born Hispanic persons experienced more day-to-day discrimination than non-U.S.–born Hispanic persons (*9*). Others have found that U.S.-born racial and ethnic minority groups have greater awareness of race-based discrimination than do non-U.S.–born persons, perhaps because race and ethnicity are experienced differently in different countries (*9*,*10*).

The findings in this report are subject to at least two limitations. First, MMP data are self-reported and subject to recall and social desirability bias. Second, the interview only captured discrimination in HIV health care settings, excluding persons not in care and not capturing other forms of discrimination.

This study underscores disparities in HIV stigma and health care discrimination experiences of Hispanic persons with HIV and the need to tailor HIV care efforts. Eliminating stigma and discrimination is a national priority and will require person-, provider-, facility-, and community-level interventions. Provider-focused trainings, policies, and practices are needed to address HIV stigma and discrimination experienced by Hispanic persons with HIV. Trauma-informed approaches to HIV care and treatment might reduce discrimination in HIV care settings by creating feelings of safety, empowerment, and trust among patients while moving beyond cultural biases and stereotypes.^¶¶¶¶^ HIV care providers should also maintain cultural and linguistic competency. Community-level interventions include supporting organizations that reflect the Hispanic population and increase access to HIV care and leveraging campaigns such as CDC’s Let’s Stop HIV Together (Detengamos Juntos el VIH).*****

Data disaggregation among Hispanic persons with HIV revealed disparities in stigma and discrimination experiences. Designing multilevel, culturally, and linguistically appropriate approaches that address stigma and discrimination, particularly among priority populations such as Hispanic persons with HIV, is key to improving care and treatment outcomes and ending the HIV epidemic.

**Summary**

**What is already known about this topic?**

Hispanic or Latino (Hispanic) persons with HIV experience disparities in health outcomes compared with other racial and ethnic groups. Eliminating stigma and discrimination, which are barriers to HIV care and treatment, is a national priority.

**What is added by this report?**

Hispanic persons with HIV commonly reported HIV stigma and health care discrimination. Among Hispanic persons with HIV, HIV stigma was highest among women (median stigma score = 35.6 of 100) and American Indian or Alaska Native persons (median stigma score = 32.7); health care discrimination was experienced more frequently by men than by women (23% vs. 18%) and by Black or African American Hispanic persons than by White Hispanic persons (28% vs. 21%).

**What are the implications for public health practice?**

Culturally appropriate efforts to reduce stigma and discrimination among Hispanic persons with HIV should consider disparities by gender and race.

## Footnotes

* As defined by the National HIV/AIDS Strategy for the United States, stigma is “an attitude of disapproval and discontent toward a person or group because of the presence of an attribute perceived as undesirable” and discrimination occurs “when unfair and often unlawful actions are taken against people based on their belonging to a particular stigmatized group.”

†
https://www.unaids.org/sites/default/files/media_asset/07-hiv-human-rights-factsheet-stigma-discrmination_en.pdf

§
https://www.whitehouse.gov/wp-content/uploads/2021/11/National-HIV-AIDS-Strategy.pdf

¶ The six demographic characteristics are gender, race, Hispanic origin, birth outside the United States, country of birth, and limited English proficiency.

**
https://www.cdc.gov/hiv/statistics/systems/mmp/projectareas.html

†† The same jurisdictions (which were sampled in 2004) have participated in MMP since 2005; however, beginning in 2015 the sampling strategy for the second stage changed to select a representative sample of adults with diagnosed HIV, regardless of care status.

§§ Personalized stigma was measured using two items: “I have been hurt by how people reacted to learning I have HIV” and “I have stopped socializing with some people because of their reactions to my HIV status.” Disclosure concern was measured using two items: “I am very careful who I tell that I have HIV” and “I worry that people who know I have HIV will tell others.” Negative self-image was measured using three items: “I feel that I am not as good a person as others because I have HIV,” “Having HIV makes me feel unclean,” and “Having HIV makes me feel that I’m a bad person.” Public attitudes were measured using two items: “Most people think that a person with HIV is disgusting” and “Most people with HIV are rejected when others find out.”

¶¶ The seven forms of discrimination included being treated with less courtesy than other people, being treated with less respect than other people, receiving poorer service than others, having a doctor or nurse act as if he or she believed they were not smart, having a doctor or nurse act as if he or she were afraid of them, having a doctor or nurse act as if he or she were better than them, and having a doctor or nurse not listen to what they were saying. Participants were asked if they experienced this never, rarely, some of the time, most of the time, or all the time.

*** Characteristics included HIV infection, gender, sexual orientation or practices, race and ethnicity, income or social class, and injection drug use. Participants could have selected more than one characteristic.

††† Race and ethnicity were measured based on Office of Management and Budget Directive No.15. Participants were asked “Do you consider yourself to be of Hispanic, Latino/a, or Spanish origin?” and “Which racial group or groups do you consider yourself to be in? You may choose more than one option.” Hispanic participants were categorized as White if they considered themselves to be White and said “no” to all other races. Asian, American Indian or Alaska Native, and Black Hispanic persons were categorized similarly. Participants who answered “no” to all races, refused to identify with all of the races, or had some combination of these were classified as “race not selected.” Participants who selected more than one race were classified as “multiple races.”

§§§ Hispanic participants were asked, “Which of the following describes your Hispanic, Latino/a, or Spanish origin? You can choose more than one.” The categories were “Mexican, Mexican American, or Chicano/a,” “Puerto Rican,” “Cuban,” and “another Hispanic, Latino/a, or Spanish origin.” Participants who selected “another Hispanic, Latino/a, or Spanish origin” or multiple Hispanic or Spanish origins (e.g., Mexican, Puerto Rican, or Cuban) were categorized as “another Hispanic origin.”

¶¶¶ Persons born in Puerto Rico or another U.S. territory were categorized as being born outside the United States for the purpose of this analysis because of differences in cultural context.

**** Participants who spoke English less than “very well” and spoke a language other than English at home were categorized as having limited English proficiency. Persons currently living in Puerto Rico were excluded from this variable because English is not the primary language spoken in Puerto Rico.

†††† 45 C.F.R. part 46.102(l)(2); 21 C.F.R. part 56; 42 U.S.C. Sect. 241(d); 5 U.S.C. Sect. 552a; 44 U.S.C. Sect. 3501 et seq.

§§§§
https://thinkculturalhealth.hhs.gov/

¶¶¶¶
https://ncsacw.acf.hhs.gov/userfiles/files/SAMHSA_Trauma.pdf

*****
https://www.cdc.gov/stophivtogether/index.html
